# Editorial: The use of real world data for regulatory purposes in the rare diseases setting

**DOI:** 10.3389/fphar.2022.1089033

**Published:** 2022-11-24

**Authors:** Viviana Giannuzzi, Violeta Stoyanova-Beninska, Virginie Hivert

**Affiliations:** ^1^ Research Department, Fondazione per la Ricerca Farmacologica Gianni Benzi Onlus, Valenzano, Italy; ^2^ Medicines Evaluation Board, Utrecht, Netherlands; ^3^ European Organisation for Rare Diseases (EURORDIS), Paris, France

**Keywords:** real world data, rare diseases, orphan medicines, medicine regulation, real world evidence

Today, Real World Data (RWD) have been recognized as an important source of information not only for public health purposes but also for scientific-health research (EU Digital Strategy eHealth, 2022; Food and Drug Administration, 2018). As an alternative or complementary to the traditional clinical research setting, such as clinical trials, evidence generated in the real-world contributes to better understanding of diseases and life-cycle of medicines. This has been acknowledged not only by public authorities (European Commission, 2018), clinicians and researchers (Polak et al.), but also by patients, who claim the need to consolidate knowledge on the economic, social, and quality of life impacts of rare diseases (Delaye et al.).

Around the world, the use of personal data including health data is ruled by data protection laws. Through these laws, citizens could control the use of their personal data. On the other hand, the diverse rules and healthcare landscape across countries result in challenges for researchers in processing and sharing data in the context of scientific research (Xiang and Cai, 2021; Sarabdeen et al., 2022), as shown in the EU (Vukovic et al., 2022) and US (Su et al., 2021).

A wider use of RWD is supported by the digitalisation of health records, use of wearable devices, sensors, smartphone applications. These tools can generate continued patient data in the home environment, as well as link different health data resources. Ferrer-Mallol et al. showed the usability of a novel digital technology tailored for Duchenne Muscular Dystrophy (DMD) patients to collect RWD and measure a clinical endpoint, Stride Velocity 95th Centile (Ferrer-Mallol et al.). Several projects have been initiated to establish data platforms as a reliable source of information (European Commission, 2021), and we will observe the use of the data from these platforms in the near future.

Despite the abovementioned concerns and limitations, the collection of health data represents a pillar in the field of rare diseases, being characterised by scarcity and heterogeneity of data dispersed across countries, making the traditional clinical research difficult and lengthy (Giannuzzi et al., 2017). One of the advantages of RWD from rare disease patients is that it may allow clinical studies enrolling fewer patients, as well shorter and less frequent hospital visits than in traditional clinical studies (Servais et al., 2022). Importantly, it has been demonstrated that rare disease patients are more predisposed to consent for data access and sharing (European Commission, 2021). This makes the use of RWD a real opportunity, despite its known drawbacks, of filling the gaps related to the lack of data.

From a regulatory perspective, RWD collected both retrospectively and prospectively and included in electronic health records, registries, claims and prescription data can provide a wide spectrum of evidence. In the literature, several studies show that this is a reality. And this has been confirmed by the articles received and published within this FRONTIERS Research Topic.

For example, RWD can provide information on disease natural history, prevalence and incidence, expected number of eligible patients and their characteristics, choice of endpoints. Such information could be supportive for decision making in different stages of orphan medicinal products life-cycle. It can be used to support the orphan designation in the pre-authorisation phase, the evaluation/refinement of the benefit/risk balance and/or maintenance of the orphan status at the marketing authorisation phase, as well as the long-term effectiveness and safety profile in the post-approval phase (Jonker et al.; Naumann-Winter et al.). It has been highlighted how realistic the applicability of RWD can be and what are the inevitable drawbacks (Naumann-Winter et al.).

A study found that about 40% of initial MAAs on orphan medicines included RWD, based on registries or hospital data (Flynn et al., 2022). For decades, RWD have been used to collect safety data, being essential to assess safety in a real-world setting rather than under the stringent conditions of the clinical trial (Cave et al., 2019). More recently, the collection of efficacy data from the “real-world” or a registry was recommended for 32% of the orphan medicines authorised in the period between 2019 and 2021 (Naumann-Winter et al.).

Polak et al. provided examples that also expanded access programmes can generate data from the real-world, even if the primary intent of such programmes remains providing non-authorised treatment to patients. RWD from these sources could supplement, rather than replace, clinical trial data for regulatory purposes (Polak et al.).

Recent publications emphasised how RWD collected within registries have a key role in increasing the knowledge of rare diseases (Jonker et al.; Mazzucato et al.; Mordenti et al.).

Furthermore, in the rapidly evolving therapeutic landscape, they can help understand unmet care needs (Mazzucato et al.). This underlines the importance of data collection for either the rarest conditions or more complex treatment settings, such as advance therapies (Naumann-Winter et al.).

The opportunity provided by RWD for regulatory purposes has been further highlighted by the COVID-19 pandemic, as they have significantly contributed to the research and development and access to COVID-19 treatments and vaccines.

Finally, it should be mentioned that RWD sources are considered one of the novel methodologies to be employed in R&D programmes, that can be “regulatorily qualified” by regulatory agencies [FDA (2022) Clinical outcome assessments (COA) qualification program; EMA (2022) qualification procedure of novel methodologies for medicine development].

Conclusively, what is still needed?

1) Quality assurance of sources. As RWD are generally not collected for research purposes (as for clinical trials), there can be concerns about data organisation, data quality, potential biases e.g., confounding factors (Jonker et al.). Such concerns lead to reluctance from regulators to rely on RWD (Mordenti et al.; Polak et al.);
2) Interoperability centered on specific data elements, ontologies, and common terminologies (Mordenti et al.);
3) Facing ethical, legal, and social issues (ELSI), as RWD processing needs to comply with specific requirements from data protection legislation.
4) Appropriate governance to define data processing and ownership (Jonker et al.; Mazzucato et al.). With regards to wearable-generated data, wearables may be vulnerable to security breaches (Servais et al., 2022).

What to do, then? To fully leverage the potential of RWD for regulatory decision-making, several actions have been proposed:

- Regulatory advice and guidance during the development of sources and tools for RWD (Polak et al.);
- Feasibility analysis and quality management with rigorous methods and validation analyses to ensure data integrity, completeness, and security, as recommended by EMA Guideline on registry-based studies (Jonker et al.), e.g., through appropriate quality indicators (Mordenti et al.);
- Use common data models complying with the FAIR (findable, accessible, interoperable, and re-usable) principles (Naumann-Winter et al.);
- Involvement of experts, including data curators and managers (Mordenti et al.);
- Engagement with patients and industry to build “fit-for-purpose” and user-friendly tools and databases for data collection (Servais et al., 2021) and with registry holders to understand opportunities and limitations provided by registry data during the regulatory procedures (Jonker et al.);
- Public-funded population-based registries (Mazzucato et al.).
